# Prefrontal photobiomodulation produces beneficial mitochondrial and oxygenation effects in older adults with bipolar disorder

**DOI:** 10.3389/fnins.2023.1268955

**Published:** 2023-10-31

**Authors:** Courtney M. O’Donnell, Douglas W. Barrett, Patrick O’Connor, F. Gonzalez-Lima

**Affiliations:** ^1^Department of Psychology, The University of Texas at Austin, Austin, TX, United States; ^2^Institute for Neuroscience, The University of Texas at Austin, Austin, TX, United States

**Keywords:** translational neuroscience, photobiomodulation, neurobehavioral conditions, bipolar disorder, older adults, broadband near-infrared spectroscopy, cytochrome-c-oxidase, brain oxygenation

## Abstract

There is growing evidence of mitochondrial dysfunction and prefrontal cortex (PFC) hypometabolism in bipolar disorder (BD). Older adults with BD exhibit greater decline in PFC-related neurocognitive functions than is expected for age-matched controls, and clinical interventions intended for mood stabilization are not targeted to prevent or ameliorate mitochondrial deficits and neurocognitive decline in this population. Transcranial infrared laser stimulation (TILS) is a non-invasive form of photobiomodulation, in which photons delivered to the PFC photo-oxidize the mitochondrial respiratory enzyme, cytochrome-c-oxidase (CCO), a major intracellular photon acceptor in photobiomodulation. TILS at 1064-nm can significantly upregulate oxidized CCO concentrations to promote differential levels of oxygenated vs. deoxygenated hemoglobin (HbD), an index of cerebral oxygenation. The objective of this controlled study was to use non-invasive broadband near-infrared spectroscopy to assess if TILS to bilateral PFC (Brodmann area 10) produces beneficial effects on mitochondrial oxidative energy metabolism (oxidized CCO) and cerebral oxygenation (HbD) in older (≥50 years old) euthymic adults with BD (*N* = 15). As compared to sham, TILS to the PFC in adults with BD increased oxidized CCO both during and after TILS, and increased HbD concentrations after TILS. By significantly increasing oxidized CCO and HbD concentrations above sham levels, TILS has the potential ability to stabilize mitochondrial oxidative energy production and prevent oxidative damage in the PFC of adults with BD. In conclusion, TILS was both safe and effective in enhancing metabolic function and subsequent hemodynamic responses in the PFC, which might help alleviate the accelerated neurocognitive decline and dysfunctional mitochondria present in BD.

## Introduction

Controlled studies of photobiomodulation (PBM) or low-level light/laser therapy (Anders et al., 2015) using specific light wavelengths from lasers or light-emitting diodes (LEDs) have demonstrated extensive cellular effects in the brain (Rojas and Gonzalez-Lima, 2011, 2017; Salehpour et al., 2018; Mitrofanis and Henderson, 2020; Cardoso et al., 2021, 2022a,b; Dos Santos Cardoso et al., 2021, 2022). Transcranial PBM has also shown therapeutic promise for various neurological conditions (reviewed in Rojas and Gonzalez-Lima, 2013; Liebert et al., 2021; Salehpour et al., 2021; Cardoso et al., 2022c,d). The beneficial use of lasers and LEDs in human neurobehavioral disorders has expanded over the last decade, including recent pilot studies in bipolar disorder (BD) (Mannu et al., 2019; O’Donnell et al., 2022). In BD, there is mounting evidence of mitochondrial dysfunction and hypometabolism in the prefrontal cortex (PFC) (reviewed in Morris et al., 2017; Andreazza et al., 2018). However, the mechanisms underlying the potential therapeutic effects of PBM in BD are not yet fully understood.

In other neurobehavioral disorders, there are more PBM studies. For example, PBM in individuals with depression has been shown to improve depressive symptoms under certain conditions (Schiffer et al., 2009; Cassano et al., 2015; Disner et al., 2016; Caldieraro and Cassano, 2019), with one study finding an anti-depressant effect size (Cohen’s d) of 0.87 (Cassano et al., 2018). Three PBM studies improved anxiety symptoms, overall severity of symptoms, and sleep in individuals with anxiety disorders (Carneiro et al., 2019; Maiello et al., 2019; Zaizar et al., 2023). PBM has also shown beneficial effects in individuals with cognitive complaint, such as improved memory, reaction time, accuracy, and increased alpha, beta, and gamma power density in electroencephalographs (EEG) (Vargas et al., 2017; Dougal et al., 2021; Nizamutdinov et al., 2021). One PBM study in individuals with mild cognitive impairment (MCI) found improved cognitive scores, sleep, anxiety, affect, and quality of life measures (Saltmarche et al., 2017), while another study on individuals with MCI showed improved executive functioning, clock drawing, immediate recall, praxis memory, visual attention, and task switching, as well as a trend of improved EEG amplitude and connectivity measures (Berman et al., 2017). Overall, PBM produces beneficial effects across various clinical neurobehavioral populations, with no reported adverse side effects.

BD is a complex and heterogeneous disorder involving severe deficits in functioning. An estimated 2.8% of U.S. adults had BD in 2017 with similar rates between males and females, affecting over 9 million people in the U.S. alone [National Institute of Mental Health (NIMH), 2017]. Eighty-three percent of individuals with BD have severe cognitive and functional impairment, which is the highest rate across all mood disorders [National Institute of Mental Health (NIMH), 2017]. Individuals with BD have a two-fold increase in cardiovascular disease and other vascular risk factors, such as decreased cerebral blood flow (CBF) (Dev et al., 2015; Toma et al., 2018), and are more susceptible to metabolic dysfunction (Mansur et al., 2013) compared to healthy, age-matched adults. BD reduces cognitive reserve and acts synergistically with other neuropathological mechanisms to accelerate aging and cognitive deterioration (Sajatovic et al., 2015). In summary, even in a euthymic state, adults with BD still present with more metabolic and functional deficits.

Transcranial infrared laser stimulation (TILS) is a form of PBM in which non-ionizing laser light at 1064-nm applied to the forehead triggers mitochondrial and cerebrovascular responses in the human brain (reviewed in Gonzalez-Lima, 2021; Wang et al., 2022). The present study examined the effects of a single 10-min session of TILS to bilateral anterior prefrontal cortex (PFC, Brodmann area 10) in older, euthymic adults with BD. Broadband near-infrared spectroscopy (bbNIRS) was used as a non-invasive method to measure changes in concentrations of oxidized cytochrome-c-oxidase ([CCO]) and differential hemoglobin ([HbD], oxygenated minus deoxygenated hemoglobin), during sham placebo and during and after TILS. CCO is a sensitive and reliable marker of neuronal activity (Wong-Riley, 1989), while HbD is an index of cerebral blood oxygenation (Tian et al., 2016). TILS to the PFC has been shown to increase [CCO] and [HbD] in healthy, young adults (Tian et al., 2016; Wang et al., 2017; Holmes et al., 2019) and older adults (Saucedo et al., 2021). The present study is the first to examine these effects of TILS on BD, and specifically in older, euthymic BD patients.

TILS-induced photo-oxidation of CCO results from the combined action of light and oxygen, and increases oxidized [CCO], which produces beneficial effects on mitochondrial oxidative energy metabolism and cerebral hemodynamics (Gonzalez-Lima and Barrett, 2014; Wang et al., 2017). Incomplete reduction of oxygen to water by CCO results in more reactive oxygen species (ROS) as a byproduct of oxidative phosphorylation (Musatov and Robinson, 2012). When ROS are produced and exceed concentrations of antioxidants, oxidative stress and damage occur (Srinivasan and Avadhani, 2012). In combination with the effects of ROS, a number of studies have shown deficits in brain energy metabolism in BD, which leads to mitochondrial-induced oxidative stress and damage (Stork and Renshaw, 2005; Yildiz-Yesiloglu and Ankerst, 2006; Brooks et al., 2009; Hosokawa et al., 2009; de Sousa et al., 2014; Kim et al., 2015). Therefore, TILS-induced enhancement of CCO’s antioxidant and metabolic properties in the PFC may help offset a portion of the metabolic impairment found in adults with BD.

Evidence of mitochondrial dysfunction in BD implicates CCO as a potentially therapeutic molecular target. Various mitochondrial diseases are comorbid with psychotic symptoms and often misdiagnosed as BD or schizophrenia (Clay et al., 2011). For example, one study found significantly smaller-sized mitochondria and a decreasing number of mitochondria from the nucleus to the outer membrane of the cell in older adults with BD (Cataldo et al., 2010). Alterations in size and distribution of mitochondria may cause deficits in plasticity, resilience, and survival (Cataldo et al., 2010). Another study found that middle-aged adults with BD had significantly lower mitochondrial DNA (mtDNA) copy number compared to healthy adults (Tsujii et al., 2019); mtDNA copy number is a biomarker for mitochondrial function and self-reported health, with a higher copy number associated with increased mitochondrial health and better self-reported health status. Lastly, another study also found significantly lower mtDNA copy number in adults with BD; however, after splitting the sample by median age, they found that the effect was driven by the older adults in their sample and was not present in young adults with BD (Fries et al., 2020). This progressive deterioration in mtDNA likely contributes to the aging-related cognitive deficits found in BD, because lower mtDNA copy number has been significantly associated with poorer cognitive performance (Tsujii et al., 2019).

Another action of TILS is the increase of [HbD], an index of cerebral oxygenation, due to increased cerebral blood flow (CBF) to the PFC (Tian et al., 2016; Wang et al., 2017). Epidemiological studies have found that reduction in CBF is a major risk factor for cognitive impairment (de la Torre, 2012, 2016). Chronic CBF reduction in an aging individual can reach a critical threshold, causing a neuronal energy crisis that accelerates oxidative stress, excessive production of ROS, impaired neurotransmitter function and other deleterious events (de la Torre, 2012, 2016). Findings of CBF in euthymic adults with BD have been inconsistent. A review of CBF studies in euthymic adults with BD found an overall decrease of CBF, which did not reach a level of significance, with most studies having small sample sizes (Toma et al., 2018). One study did not find any significant differences in CBF in euthymic adults with BD (Dev et al., 2015); however, another study found decreased CBF in euthymic adults with BD (Culha et al., 2008).

Changes in both oxidized [CCO] and [HbD] have been measured non-invasively before, during and after TILS using bbNIRS in healthy young and older adults. Five studies have found a significant increase in levels of oxidized [CCO] and/or [HbD] within minutes of TILS in the right PFC, which continued to increase for 5 min after TILS (Tian et al., 2016; Wang et al., 2017; Holmes et al., 2019; Pruitt et al., 2020; Saucedo et al., 2021). Another study used two-wavelength functional near-infrared spectroscopy (fNIRS), which is similar to bbNIRS but it cannot capture changes in CCO (Tian et al., 2016). In healthy young adults, TILS produced significant increases in [HbD] measured by fNIRS, which continued up to 8 min after TILS (Tian et al., 2016). However, healthy older adults have a diminished [HbD] response as compared to younger adults (Saucedo et al., 2021).

Based on these studies, we used bbNIRS to non-invasively evaluate mitochondrial oxidative metabolism in the PFC of BD patients *in vivo* by measuring changes in oxidized [CCO] during sham placebo, and during and after TILS. In addition, bbNIRS served to evaluate cerebral blood oxygenation via changes in [HbD] during sham placebo, and during and after TILS. We hypothesized that TILS would increase both oxidized [CCO] and [HbD] in the PFC of older BD patients, with a weaker effect on [HbD] response as compared to sham.

## Methods and materials

### Participants and study design

Participants (*N* = 15, 9 females) were recruited through a local outpatient clinic, the Seton Mind Institute in Austin, Texas. Resident psychiatrists reviewed patient charts to evaluate if they met inclusionary criteria for the study. To inform the clinic of a participant’s enrollment in the study, a one-page document was emailed to the director of the clinic that included date(s) of participation and participants’ name. Participants received $25 and a parking pass upon completion of the session. In this study, all participants received active TILS treatment after receiving a within-subject sham placebo. All experimental procedures were reviewed and approved by the Institutional Review Board of the University of Texas at Austin and complied with NIH guidelines on human subject research. Table 1 shows an overview of the study visit.

**Table 1:** Timeline of study design.

Informed consent Medical history questionnaire
Pre-laser broadband near-infrared spectroscopy (bbNIRS) measurement (2-min baseline, 5-min pre-laser sham) bbNIRS and TILS administration (10 min) Post-laser bbNIRS measurement (5 min)

### Inclusionary criteria

Participants who met the following inclusionary criteria were enrolled in the study: (1) age 50 or older, (2) diagnosed with either Bipolar I or II or mixed, (3) no history of cerebrovascular accident or traumatic brain injury, (4) not actively in a mood episode, i.e., euthymic, and (5) no changes in medication for their bipolar diagnosis within 30 days of starting the study. Criterion 3 mitigated any potential bias as to the source of their functional impairment. Criteria 4 and 5 ensured that individuals were both euthymic and on a steady regimen of medication to eliminate neurocognitive impairment that may be present in active mood states or due to an individual’s response to a new medication or dosage.

### Materials

Participants completed a medical questionnaire that asked specifically about their bipolar diagnosis, number of psychiatric hospitalizations, number of manic or hypomanic episodes, suicide attempts, any comorbid psychological or psychiatric disorders, medical conditions, and current prescribed medication. Written informed consent was obtained from each participant prior to the start of the session.

### TILS administration

Administration of TILS consisted of the application of infrared light at 1064 nm using a well-collimated flat-top laser (CytonPro-5000, CytonSys Inc., Austin, Texas, United States). The TILS device was operated using the standard operating procedure approved by the University of Texas at Austin Laser Safety Office. All individuals who operated the laser have completed the laser safety class through the Environmental Health and Safety office of the University of Texas at Austin. Both participants and researchers wore protective eyewear for the entirety of the TILS session.

**Figure 1:**
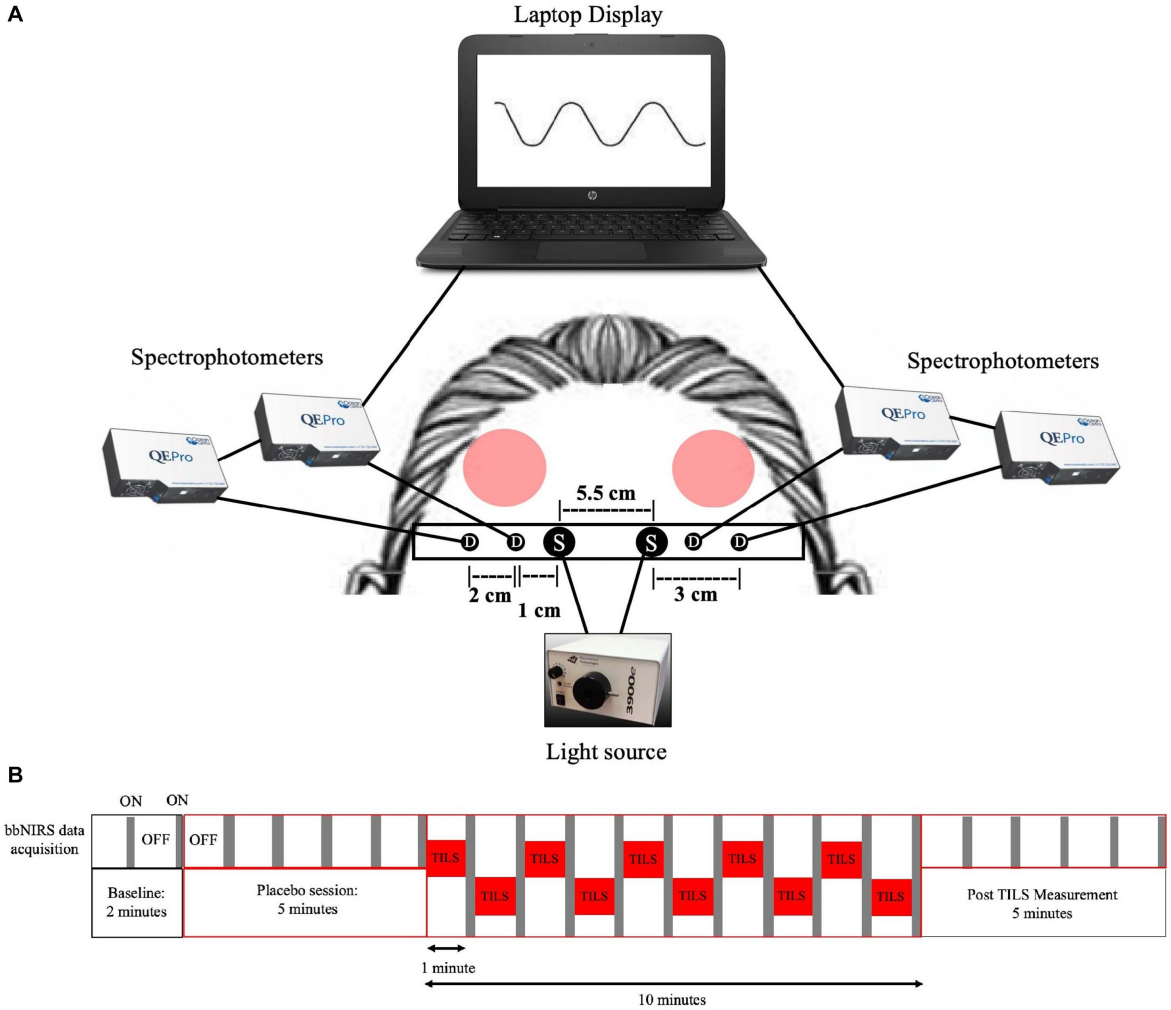
Four-channel bbNIRS system and data acquisition timeline. **(A)** Four-channel bbNIRS set-up. Participants sat in a slightly reclined optometry chair with a headrest. This bbNIRS system contained two sources and four detectors, with an elastic band superior to the eyebrows that wrapped around the back of participants’ head and was secured to the headrest to minimize introduction of movement artifacts (“S” is for light sources and “D” is for detectors). Optical cables were secured to the band and connected to four separate spectrophotometers, one for each detector. Data was displayed on a laptop computer, which was connected to the spectrophotometers. Protective eyewear was worn by the participant and laser administrator. Participants received TILS treatment (red circles noting “TILS”) to the bilateral anterior PFC regions. The treatment consists of one-minute stimulations, alternating between the right and left anterior PFC (bilateral Brodmann area 10). **(B)** Temporal sequence of alternating bbNIRS recordings and TILS administration. In order to avoid interference between the near-infrared light source of bbNIRS and TILS, bbNIRS measurements were taken for 5 s after each minute of TILS. This bbNIRS sampling protocol also occurred during baseline, pre-TILS and post-TILS.

The laser was emitted from a handheld probe, with a measured laser beam area of 13.6 cm^2^. The laser wave was continuous (CW), and the power output was 3.4 W. The irradiance (power density) was 250 mW/cm^2^ (3,400 mW/13.6 cm^2^ = 250 mW/cm^2^). The fluence dose (energy density) was 75 J/cm^2^ (0.25 W/cm^2^ × 300 s = 75 J/cm^2^) per site. Therefore, the total forehead fluence dose per session was 150 J/cm^2^ (75 J/cm^2^ × 2 forehead sites = 150 J/cm^2^). The forehead sites corresponded to the frontal polar Fp1 (left) and Fp2 (right) standard electrode placement sites used in human EEG (Wang et al., 2021). Active laser exposure time was a total of 10 min and alternated each minute between the right and left forehead sites to target the anterior PFC (Brodmann area 10) (Figure 1A). The handheld probe also emitted a visible 650 nm red guiding light, with a power density of 0.16 mW/cm^2^. Since the infrared laser light was invisible, the red light was used as an aiming light during TILS and sham placebo periods. The distance between the participants’ forehead and probe was 5 cm.

### Broadband near-infrared spectroscopy

Changes in oxidized [CCO] and [HbD] were measured via a custom-made 4-channel bbNIRS system, as detailed in our previous studies (Wang et al., 2017; Saucedo et al., 2021). This bbNIRS system provided a non-invasive technique that allowed for *in vivo* monitoring of changes of tissue chromophore concentrations and contained two light sources and four detectors. Sources transmitted broadband white light, while detectors measured light absorption across a spectrum of wavelengths, which was used to calculate concentration changes from baseline of chromophores.

Figure 1A shows the 4-channel (2 cables from 2 light sources and 4 cables from 4 spectrophotometer detectors) bbNIRS experimental setup. The spectral range of the light sources (labeled “S” in Figure 1A) was 400–1,000 nm (Illumination Technologies, Inc., Liverpool, NY, USA). A low-pass filter with a cut-off frequency of 1,000 nm attached to the light box source served to minimize heat from the system. The four bbNIRS spectrophotometers had a spectral range of 735–1,100 nm (QE-Pro, Ocean Optics Inc., Orlando, FL, USA). An elastic band held the cables of the sources and detectors in place on the participant’s forehead. A bundle of four detectors (“D” in Figure 1A) relayed the returning light via fiber-optic cables to the bbNIRS spectrophotometers, which measured the intensity of light at each point on the spectral range. A laptop computer acquired, displayed, and saved all data.

The timeline of bbNIRS measurement and TILS administration is shown in Figure 1B. The bbNIRS signal was sampled for 5 s once a minute across the 22-min session. Specifically, for the TILS session, TILS was active for 60 s and then turned off, bbNIRS data acquisition was then turned on and acquired for 5 s and then turned off, before TILS was turned back on. This interleaved acquisition method was used to avoid any potential interference between the light source of the bbNIRS system and the infrared light of TILS. This acquisition method was also used in the baseline, sham placebo, and post-TILS measurements to maintain consistency across the entire session. The bbNIRS session consisted of a 2-min baseline epoch, 5-min sham placebo epoch, 10-min TILS epoch, and 5-min post-TILS epoch. The sham placebo epoch used the same conditions as the TILS epoch, except that the laser was turned off and only the guiding red light was on.

Methodological limitations of bbNIRS do not allow for the measurement of an absolute value of concentration for any of the chromophores, so a change of concentration (Δ) from a two-minute baseline was calculated. The bbNIRS data was preprocessed by removing outliers (+/− 2.5 standard deviations) within each subject. Once outliers were removed, bbNIRS data was then detrended by generating a least-squares regression line of the sample’s pre-TILS data (first 5 min). This was done to remove any linear trends from artifacts unrelated to the TILS effects, such as baseline drift due to subject movement causing optodes to shift relative to the forehead, blood pressure variation, and instrumental instability such as thermal changes in light source and sensors (Zhao et al., 2018). Deviations from this regression line were then subtracted from each time point during TILS and post-TILS. Detrended temporal values of changes in concentrations of oxidized cytochrome-c-oxidase (Δ[CCO]) and differential hemoglobin (oxygenated hemoglobin minus deoxygenated hemoglobin, Δ[HbD]) were then averaged across the sample. Given the bilateral stimulation, hemispheric changes were not examined separately and were averaged together for each individual. To evaluate the effects of TILS, with respect to pre-TILS and post-TILS, paired-sample t-tests were calculated for Δ[CCO] and Δ[HbD] between pre-TILS and TILS, TILS and post-TILS, and pre-TILS and post-TILS. All *p*-values were corrected for multiple comparisons using the sharper Bonferroni procedure (Hochberg, 1988) and differences with a corrected *p* < 0.05 were regarded as statistically significant. All statistical analyses were completed using the statistics program R (R Core Team, 2017).

## Results

All 15 participants, with an average age of 62.5 (SD ± 9.6), completed the study. Table 2 shows the demographic and clinical data of participants. No adverse side effects were reported and no sex effects were noted.

**Table 2:** Participant demographic and clinical characteristics.

	Sample (*N* = 15)
**Mean age (y)**	62.53 ± 9.58
**No. of females (%)**	9 (60%)
**Ethnicity**	No. (%)
Caucasian	11 (73%)
Mixed	2 (13%)
Hispanic	1 (6%)
Asian	1 (6%)
**Education**	No. (%)
High school degree	5 (33%)
College degree	5 (33%)
Master’s degree	5 (33%)
Sample average (years)	16.50
**Diagnosis**	No. (%)
BD I	3 (20%)
BD II	9 (60%)
Mixed/Unspecified	3 (20%)
**Avg. # of hospitalizations (lifetime)**	2.25
**Avg. # of manic episodes (lifetime)**	6.23
**Avg. # of hypomanic episodes (lifetime)**	32.69
**Avg. # of suicide attempts (%)**	14 (30%)
**Medical history**	No. (%)
Depression	8 (50%)
Anxiety	6 (38%)
ADHD	2 (13%)
OCD	1 (6%)
**Medication category**	No. (%)
Anticonvulsant	13 (87%)
Antidepressant	8 (50%)
Antipsychotic	6 (38%)
Mood stabilizer (Lithium)	1 (6%)

### Time courses of TILS-induced Δ[CCO] and Δ[HbD]

**Figure 2:**
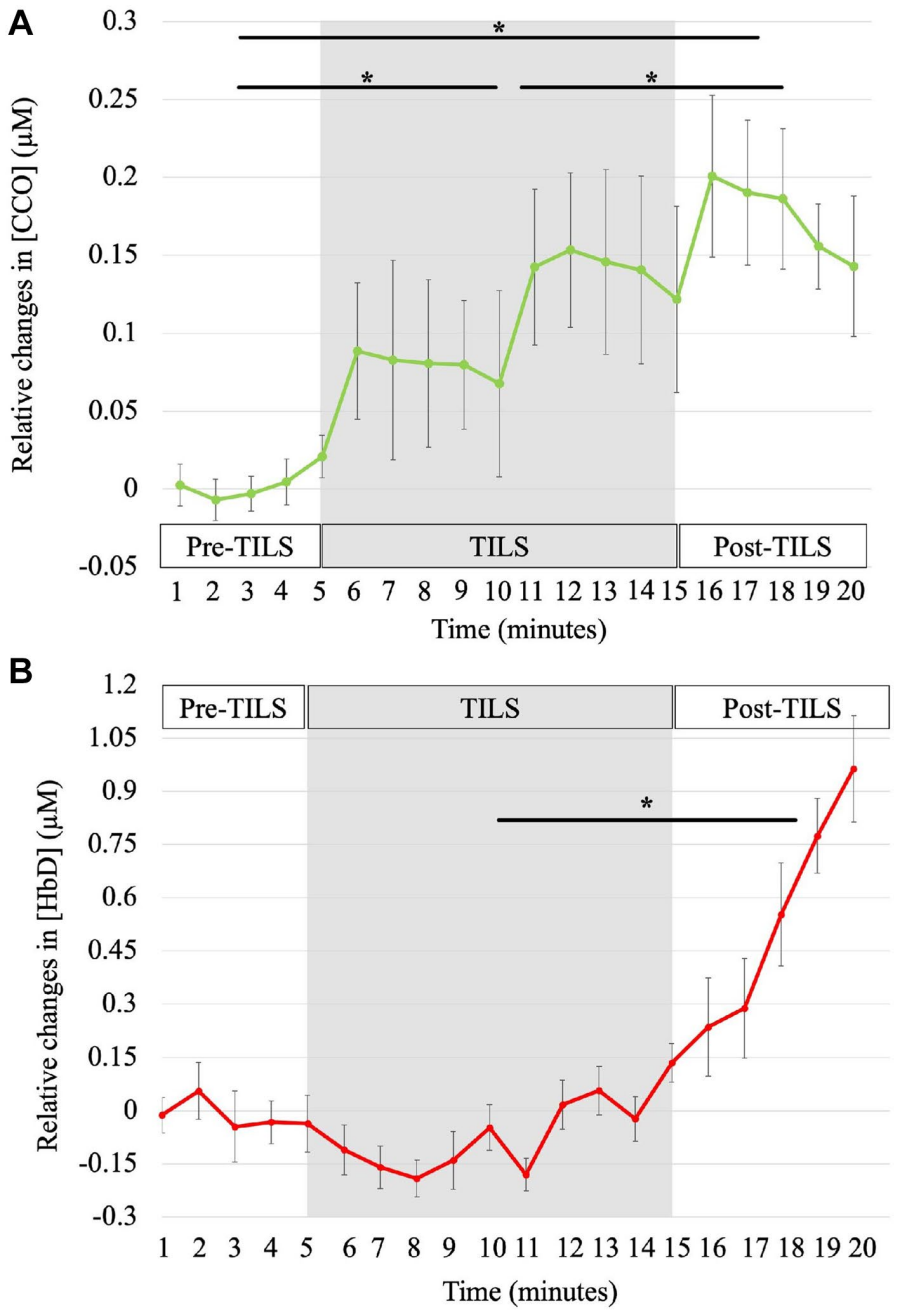
Time courses of TILS-induced Δ[CCO] and Δ[HbD]. The bbNIRS data showed changes from baseline for every minute during 5 min of pre-TILS measurement under sham-placebo conditions, 10 min of active TILS, and 5 min of post-TILS measurement. The shaded region represents active TILS. Standard errors bars are shown with asterisks noting significant differences between parts. **(A)** Effect of TILS on Δ[CCO]. [CCO] significantly increased across all three parts of the bbNIRS session, *corrected *p* < 0.003. **(B)** Effect of TILS on Δ[HbD]. [HbD] significantly increased between TILS and post-TILS, *corrected *p* = 0.04.

Figure 2A shows the progressive bilateral changes of oxidized [CCO], calculated as the change from a two-minute baseline, for pre-TILS sham placebo (minutes 1–5), TILS (minutes 6–15) and post-TILS measurements (minutes 16–20). Figure 2B shows the time course of bilateral changes of [HbD] calculated as change from a two-minute baseline.

For relative changes of [CCO], paired-sample t-tests showed significant differences between all three conditions of bbNIRS measurement (pre-TILS, TILS, and post-TILS). To account for multiple comparisons, all reported *p*-values have been corrected by sharper Bonferroni adjustment. Group means (*M*) and standard errors (*SE*) of micromole concentration changes from baseline in pre-TILS [CCO] (*M* = 0.004, *SE* = 0.01) were significantly lower compared to [CCO] changes during the TILS period (*M* = 0.11, *SE* = 0.05), *t*(14) = −4.21, corrected *p* = 0.003. Pre-TILS [CCO] (*M* = 0.004, *SE* = 0.01) and post-TILS [CCO] (*M* = 0.18, *SE* = 0.04) changes were also significantly different, with post-TILS [CCO] having greater changes in [CCO] from pre-TILS, *t*(14) = −4.36, corrected *p* = 0.003. In addition, changes in [CCO] significantly increased from the TILS period (*M* = 0.11, *SE* = 0.05) to post-TILS (*M* = 0.18, *SE* = 0.04), *t*(14) = 3.80, corrected *p* = 0.002.

For relative changes of [HbD], paired-sample t-tests showed a significant difference between TILS (*M* = −0.06, SE = 0.06) and post-TILS (*M* = 0.56, SE = 0.14), *t*(14) = −2.25, corrected *p* = 0.04. There were no significant differences between pre-TILS (*M* = −0.01, SE = 0.07) and TILS, *t*(14) = 0.20, corrected *p* = 0.85, or pre-TILS and post-TILS, *t*(14) = *t* = −1.31, corrected *p* = 0.10. Overall, TILS resulted in significantly increased oxidized [CCO] between pre-, during and post-TILS, with [HbD] significantly increasing between TILS and post-TILS. Results from this study are consistent with the hypotheses of TILS inducing increases in [CCO] and [HbD] in the PFC of older BD patients.

## Discussion

This study used bbNIRS to capture the hemodynamic and metabolic response of a single 10-min session of TILS to bilateral PFC in older, euthymic adults with BD. Euthymic and symptomatic individuals with BD both present with deficiencies in brain energy metabolism and oxygenation (Stork and Renshaw, 2005; Yildiz-Yesiloglu and Ankerst, 2006; Brooks et al., 2009; Hosokawa et al., 2009; de Sousa et al., 2014; Kim et al., 2015), particularly in the PFC (Frey et al., 2007). Older adults with BD have deficiencies in markers of oxidative stress, mitochondrial dysfunction (Fries et al., 2020), and a two-fold increase in cardiovascular disease and other vascular risk factors (Toma et al., 2018). With [CCO] as a marker of metabolic mitochondrial function and [HbD] as a measure of cerebral blood oxygenation, this study found beneficial effects of TILS in this population, in terms of increased oxidized [CCO] during and after TILS and increased [HbD] after TILS. This study confirmed previous NIRS studies showing that *in vivo* TILS increases oxidized [CCO] and/or [HbD] in the human brain (Tian et al., 2016; Wang et al., 2017; Holmes et al., 2019; Pruitt et al., 2020; Saucedo et al., 2021).

### TILS-induced Δ[CCO]

Increases in oxidized [CCO] correspond to an increase in metabolic energy produced by oxidative phosphorylation (Gonzalez-Lima and Barrett, 2014), making CCO a sensitive and reliable biomarker of neuronal activity and cellular energy metabolism (Wong-Riley, 1989). This study found that TILS-induced increases in oxidized [CCO] were present within the first minute of stimulation and continued until 5 minutes after TILS was turned off. Within subjects, this photo-oxidation time course was highly significant and amounted to 27.5 times greater change in oxidized [CCO] concentration during TILS relative to sham, and to 45 times greater concentration change post-TILS compared to sham.

This increase in oxidized [CCO] caused by TILS upregulates mitochondrial respiration, as explained in detail before (Wang et al., 2017; Gonzalez-Lima, 2021). Briefly, TILS delivers photons to CCO (Complex IV in the electron transport chain, ETC), which are absorbed by the copper ions inside CCO that donate their electrons to oxygen, thus making CCO photo-oxidized; as this happens, CCO pumps four protons (H+) into the mitochondrial intermembrane space and releases nitric oxide (NO) from its catalytic pocket, allowing CCO to catalyze the reduction of oxygen to water; then, the four protons (H+) released into the intermembrane space move into the negatively-charged mitochondrial matrix by electromotive force, driving the phosphorylation of adenosine diphosphate to adenosine triphosphate by Complex V. Any oxygen not fully reduced to water by CCO becomes a reactive oxygen species (ROS). This PBM mechanism presumably can enhance oxidative phosphorylation and reduce ROS-derived oxidative damage in older adults with BD.

Individuals with BD have impaired brain oxidative energy metabolism, decreased antioxidant enzymes, increased lipid peroxidation, and an overproduction of ROS (Stork and Renshaw, 2005; Berk et al., 2011). The combination of an overproduction of ROS and decreased production of antioxidants leads to oxidative stress and damage (Srinivasan and Avadhani, 2012). An accumulation of oxidative damage leads to apoptosis and an aggregation of oxidized proteins, which may result in the impairment of mood-stabilizing mechanisms and the exacerbation of the pathophysiology of BD (Berk et al., 2011). A review paper examined the specific effects of oxidative stress and damage on CCO in the ETC (Musatov and Robinson, 2012). They reported that CCO is a consistent producer and target of ROS because it is the only ETC complex that reacts directly with oxygen during oxidative metabolic processes. The involvement of CCO in oxidative stress is associated with increased ROS and cellular toxicity (Srinivasan and Avadhani, 2012). Given the susceptibility of CCO to oxidative damage and the known oxidative stress in BD, increasing oxidized [CCO] would promote the reduction of oxygen to water, instead of promoting a partial reduction of oxygen that produces ROS. This action of TILS might presumably alleviate ROS-induced oxidative stress in the brains of older adults with BD.

Importantly, a pilot study of depressed individuals with BD found significantly lower oxidized [CCO] changes in the anterior PFC (Brodmann area 10) correlating inversely with depression severity (Holper et al., 2019). Our findings showed that TILS produced significant increases from baseline in oxidized [CCO] in the anterior PFC, and thus TILS might enhance, even if temporarily, brain energy metabolism while decreasing oxidative stress in older, euthymic patients with BD.

### TILS-induced Δ[HbD]

Cerebral blood flow (CBF) is the blood supply to the brain in a given period of time and is a key metric of brain health (Toma et al., 2018). In this study, [HbD] was used as an index of CBF and oxygenation, with increasing [HbD] reflecting increases in oxygenated hemoglobin and decreases in deoxygenated hemoglobin. The majority of studies of BD depression and mania have found lower PFC CBF, but CBF research in euthymic BD is limited, with one meta-analysis finding widespread hypoperfusion that did not reach a level of significance (Toma et al., 2018). These non-significant findings of CBF in euthymic adults with BD may be attributed to limited sample sizes and methodological inconsistencies and should be further explored in future studies.

This study confirmed previous studies showing that *in vivo* TILS increases PFC oxygenation in the human brain (Tian et al., 2016; Wang et al., 2017, 2018; Holmes et al., 2019; Pruitt et al., 2020; Saucedo et al., 2021). For example, young healthy adults showed a significant increase in [HbD] within 2 min of TILS which continued to increase 5 min after TILS was turned off (Tian et al., 2016; Wang et al., 2017). However, in older healthy adults, their hemodynamic responses were delayed (Saucedo et al., 2021). The results of our study in older adults with BD revealed an even greater delay in increases in [HbD], with significant increases in [HbD] between TILS and post-TILS. In comparing to these previous studies, this difference could be due to a number of factors. First, participants in this study were older adults, a population which may show a delayed hemodynamic response. The Centers for Disease Control reported that over half of adults in the U.S. are diagnosed with cardiovascular disease and have poor vascular health (Benjamin et al., 2019); also, cardiovascular health and cognitive brain health are closely linked (Van der Velpen et al., 2017). Further, symptomatic individuals with BD have a two-fold increase in cardiovascular disease and other vascular risk factors, such as cerebral hypoperfusion or decreased CBF, compared to healthy age-matched adults (Dev et al., 2015; Toma et al., 2018). Second, the delay in [HbD] response could be due to the presence of a psychiatric disorder. It is well established that there is severe hypofrontality and impaired connectivity in the PFC of euthymic adults with BD (Townsend et al., 2010; Schecklmann et al., 2011; Cremaschi et al., 2013). This impaired PFC functioning could result in the relative delay in cerebral hemodynamic response to TILS. Lastly, the majority of individuals with BD have experienced a major depressive episode. Adults with BD in an active mood state show decreased CBF bilaterally in PFC regions (Bhardwaj et al., 2010), as well as a trend of PFC hypometabolism (Schecklmann et al., 2011). In summary, given the deficits in baseline hemodynamic functioning of the PFC in older adults with BD as compared to healthy, young adults, the delay in response to TILS can be attributed to the participants’ older age, psychiatric diagnosis, and prior history of depression.

### Neurovascular coupling

In addition, the delayed significant increase of [HbD] induced by TILS likely also reflects the impaired neurovascular coupling of the PFC in BD (Toma et al., 2018). Neurovascular coupling involves paired responses that link neural metabolic activity to vascular hemodynamic activity (Huneau et al., 2015). The abnormal neurovascular coupling seen in BD is also present in aging adults and in neurodegenerative diseases (Tarantini et al., 2017). Wang et al. (2017) examined TILS-induced metabolic and hemodynamic coupling in order to quantify the coupling strength between TILS-enhanced cerebral metabolism and hemodynamic response. They found that young, healthy adults had a strong neurovascular coupling between the change of [CCO] and [HbD] during and post-TILS, with a coefficient of determination of R^2^ = 0.96. We conducted the same analysis in our older adults with BD and found a much weaker coupling between change of [CCO] and [HbD], during and post-TILS, with R^2^ = 0.34. This analysis suggests that neurovascular coupling is not as closely regulated in older adults with BD, with the metabolic response (CCO) happening quicker than the hemodynamic response (HbD) to TILS. This abnormal neurovascular coupling is likely a reflection of both the participants’ age and their diagnosis. Future studies should examine this weaker coupling in a larger sample of older adults with and without BD and assess if multiple sessions of TILS can counteract this deficit.

### Comparison of TILS with non-invasive magnetic and electrical stimulation

Medications are effective in treating BD in some patients, but they are not clinically effective in everyone. Recently, various forms of brain stimulation have been explored as potential treatments for BD and other mental, psychiatric, and cognitive disorders. The most commonly-used non-invasive techniques for treating these disorders are repetitive transcranial magnetic stimulation (rTMS) and transcranial direct current stimulation (tDCS) (reviewed in Camacho-Conde et al., 2023). In rTMS, short electromagnetic pulses are used to stimulate superficial cortical brain regions, which modulates the excitability of the tissue. In tDCS, a low level of current is introduced to the scalp, which can target either a diffuse area of tissue or a relatively focused one via high-definition tDCS. Like TILS, these are safe, non-invasive methods of delivering energy to the brain *in vivo*, targeting cortical brain regions for the purpose of brain stimulation. In rTMS and tDCS, this energy is in the form of electrical currents, induced by magnetic fields or electrodes respectively, while in TILS, the energy is in the form of photons of a particular wavelength. All three methodologies involve targeting frontal cortex for the purpose of improving cognition. While rTMS and tDCS directly modify the electrical excitability of cellular membranes, TILS operates via a bioenergetic effect, involving the photonic oxidation of CCO, which evokes a hemodynamic response (Tian et al., 2016; Wang et al., 2017, 2018; Saucedo et al., 2021).

Reviews of the use of rTMS and tDCS in the treatment of bipolar disorder found that both techniques were clinically effective (Dondé et al., 2017; Nguyen et al., 2021), and our recent pilot study demonstrated that TILS was effective at improving cognition in older individuals with bipolar disorder (O’Donnell et al., 2022). Neuroplasticity can be induced by both rTMS (Lenz et al., 2016) and tDCS (Ranieri et al., 2012), and we recently found evidence of increased neuroplastic changes in the rat brain after a single treatment of TILS (Wade et al., 2023). The induction of neuroplasticity by these methodologies may be one key mechanism by which they have a therapeutic effect in bipolar disorder (Zamar et al., 2022; D'Urso et al., 2023). rTMS, tDCS, and TILS also show promise in treating age-related cognitive decline (Vargas et al., 2017; Cai et al., 2019; Phipps et al., 2021). Since neuroplasticity decreases with age (Park and Bischof, 2013), it is possible that the induction of neuroplasticity by these methodologies may protect against neurocognitive aging and any disorder involving brain hypometabolism, which includes bipolar disorder (Toma et al., 2018). In general, though their neurobiological mechanisms differ, rTMS, tDCS, and TILS have all been used to improve various forms of cognition and treat a variety of mental disorders (Montazeri et al., 2022; Camacho-Conde et al., 2023).

### Limitations

There were several limitations in this study. First, BD is a clinically heterogeneous and complex disorder, and the sample size in this study was small (N = 15). This study did not limit BD type, with most participants (n = 9) being diagnosed with BD II. Thus, our results may be more relevant to BD II. Participants were only examined during a single session, which is a limitation when assessing clinical populations. Given the complexity of BD, it is important to understand the variability across participants and the need for individualized dosing of TILS. This study used the same TILS parameters of previous studies for irradiance (power density, 250 mW/cm^2^) and similar fluence dose (energy density per site, 75 J/cm^2^), which have been shown to be effective in healthy young and older adults (Barrett and Gonzalez-Lima, 2013; Blanco et al., 2017a,b; Saucedo et al., 2021), adults with elevated depressive symptoms (Disner et al., 2016) and older adults with subjective cognitive complaint (Vargas et al., 2017). A higher power and/or energy density may be needed to produce optimal effects in the bipolar population.

### Future directions

To benefit from bbNIRS measures in adults with BD, future studies should assess if [CCO] and [HbD] responses to TILS can predict cognitive task performance. This is an important factor in understanding molecular target engagement to assess the effects of an intervention. Future studies should measure different forms of target engagement, in hopes of measuring the response to TILS and categorizing patients as a responder or non-responder. For example, functional near-infrared spectroscopy (fNIRS) might be used to quantify changes in [HbD] in the entire PFC, both pre- and post-TILS. Patients that significantly increased their [HbD] from baseline would be enrolled in the study. Based on the present bbNIRS study, it is hypothesized that no one will be entirely a “non-responder,” but that there will be varying degrees of “responders.” It is also anticipated that “lower-responders” would need an increased dose of TILS, whereas “higher-responders” might require doses of TILS comparable to those found to significantly improve cognitive performance in healthy participants (Barrett and Gonzalez-Lima, 2013; Vargas et al., 2017; Blanco et al., 2017a,b; Holmes et al., 2019). This type of analysis would provide insight into understanding individualized dosing and potentially generating a dose–response curve for TILS in BD. Future studies in adults with BD should assess multiple sessions of TILS and examine if treatment effects are still present several weeks after TILS. Future studies should also focus on increasing sample size and expanding the age range of adults with BD.

## Conclusion

This study was the first to assess the effects of a single session of TILS in older adults with BD, and more work is needed to characterize the duration and frequency of TILS treatment. Once TILS has been validated to significantly increase [CCO] or [HbD] in a larger sample of adults with BD, randomized controlled trials will be needed. Elucidating the long-term effects of TILS in older adults with BD would provide additional insight into the clinical application and necessary dosing of TILS. Lastly, future studies should incorporate other imaging modalities, along with bbNIRS. Many research studies on euthymic adults with BD have been conducted using positron emission tomography (Brooks et al., 2009; Hosokawa et al., 2009; Sargent et al., 2010; Haarman et al., 2014) and magnetic resonance spectroscopy (Brady et al., 2012; Sosa-Moscoso et al., 2022), with studies using arterial spin labeling (Dev et al., 2015; He et al., 2019) gaining more prevalence (reviewed in Cremaschi et al., 2013; Toma et al., 2018). In conclusion, TILS offers a novel way to enhance cerebral metabolic and hemodynamic properties in older adults with BD.

## Data availability statement

The raw data supporting the conclusions of this article will be made available by the authors, without undue reservation.

## Ethics statement

The studies involving humans were approved by the Institutional Review Board of the University of Texas at Austin. The studies were conducted in accordance with the local legislation and institutional requirements. The participants provided their written informed consent to participate in this study.

## Author contributions

CO’D: Conceptualization, Formal analysis, Investigation, Methodology, Visualization, Writing – original draft, Writing – review & editing. DB: Conceptualization, Formal analysis, Methodology, Project administration, Supervision, Visualization, Writing – original draft, Writing – review & editing. PO’C: Investigation, Methodology, Software, Writing – review & editing. FG-L: Conceptualization, Formal analysis, Funding acquisition, Methodology, Project administration, Supervision, Writing – original draft, Writing – review & editing.
